# Singular Effects of Ipecacuanha

**Published:** 1843-08

**Authors:** Felix Robertson

**Affiliations:** Nashville, Tennessee


ART. III.—*Singular effects of Ipecacuanha.*—A Case reported to the Medical Society of Tennessee. By FELIX ROBERTSON, M.D., of Nashville, Tennessee.

The summer and fall of 1823 and '24, were extremely sickly, fevers of a high grade prevailed throughout this region of country. The necessary labor and exposure in attending to an extensive country practice had nearly worn me down, had greatly reduced my strength. About the last of October while putting up a dose of powdered ipecacuanha, I was suddenly seized with a violent attack of asthma, attended with the most distressing dyspnœa and oppression at the precordia. Bleeding and active cathartics relieved the attack in five or six days. This kind of attack was repeated several times before I was confident they were brought on by breathing the dust of ipecacuanha. This however was at last reduced to a certainty, but not before my weakened health, &c., laid me liable to attacks from other exciting causes.

In November 1825 I left on business for Texas, but hoping the change of climate might have a good effect on my greatly enfeebled health. I of course was exposed in such a trip to many causes that might have been expected to bring on attacks of the asthma, such as sleeping in the open air, &c., &c. I was absent on that trip six months, and returned to Tennessee in apparently better health than I had enjoyed for several years past, not having had the slightest attack of the disease during my absence. A few weeks after resuming the business of my profession, I felt unwell, with deranged state of the stomach, and thought it advisable to take an emetic—wishing to take a mild safe article I concluded to take wine of ipecac. and never dreamed of any unusual bad effect it could have on me. From the moment I swallowed it I felt in the throat and stomach a sensation totally indescribable, but as intolerable to be borne, (if life could have been sustained under it), as if I had taken a drink of melted lead. It was so overpowering that I was unable to think of any method of relief, but in the most distracting agony leaped out of bed and rolled over the floor from side to side of the room. At length I was urged to drink copiously of warm water, which produced vomitng and some mitigation of my intense suffering. This distress slowly subsided and settled into one of my worst attacks of asthma. I was again subject to these attacks from the smallest particle of ipecac. breathed, or the fumes of burning sulphur; and the exciting causes seemed to multiply with every return of the disease, and my health became so feeble that I was seldom able to attend to the calls of my profession, and was compelled entirely to give up country business. I at length determined to try the effect of reducing the thickness of my clothes, to sleep on a hard mattress under as light covering as possible, and avoid if possible getting into a profuse perspiration, which always appeared to invite an attack. At the same time I determined to use snuff for the purpose of lessening the sensibility of the nasal membrane, as my attacks seemed to commence generally with a feeling of irritation of that membrane, which rapidly extended itself to the bronchia and all its ramifications. My attacks from this period became lighter, of shorter duration and with increased intervening intervals, and in the course of a year I got entirely clear of them, except occasionally some dyspnœa from accidentally coming in contact with ipecac. from stepping into an apothecary's shop shortly after the article had been handled.

For some seven years past I have not had a severe attack, and my general health has greatly improved. About three years since I was induced to put my tongue to some liquid in a vial that was believed to be wine of ipecac,, a patient wishing to take it if I knew it to be that article. I had of course not forgotten the terrible effects of the emetic I had once taken, but believed I could taste it without any injury arising from it. In an instant that peculiar, burning, indescribable sensation was felt in the mouth, which rapidly extended itself to the fauces and down the throat and bronchia, and for two hours I suffered greatly, and had considerable dyspnœa, all of which gradually disappeared; and while I retain my understanding no such intimacy will ever be repeated with the fated article. What makes the case more remarkable is that ipecac. had always been with me a favorite article of medicine, and no one handled it freer or with less reserve up to the very day it committed such an unprovoked and desperate attack on me.

I observed in some of my attacks a very curious sequela. When expectoration became free and mucus was formed in great quantities by the mucous membrane of the bronchia, in the morning, after resting through the night from the cough, expectoration would commence freely on stirring about a little, and mouthsfull would be thrown up, which any person at first sight would have pronounced to be a mass of small, nearly transparent worms—on close examination I discovered it was thickened mucus which had collected in the small ramifications of the bronchial tubes during sleep, and was actually discharged so as to be real casts of those tubes. This would sometimes be thrown up in such quantities, in the morning, that it really surprised me that sufficient air could have been passed through the lungs for the purposes of life while sleeping.

